# No association of a Vascular endothelial growth factor A (VEGFA) gene polymorphism with pre-eclampsia among pregnant women in Uganda

**DOI:** 10.1186/s12864-023-09213-8

**Published:** 2023-03-20

**Authors:** Sheila Nabweyambo, Stephen Kanyerezi, John H.-O. Petterson, Fred Ashaba Katabazi, Alfred Ssekagiri, Savannah Mwesigwa, Gerald Mboowa, Faith Nakazzi, Annette Keesiga, Moses Adroma, Freddie Bwanga, Naomi McGovern, Obondo James Sande, Annettee Nakimuli

**Affiliations:** 1 Department of Medical Microbiology, School of Biomedical Sciences, College of Health Sciences, Makerere University, Kampala, Uganda; 2 The African Center of Excellence in Bioinformatics and Data-Intensive Sciences, Infectious Diseases Institute, Makerere University, Kampala, Uganda; 3 Department of Immunology and Molecular Biology, School of Biomedical Sciences, College of Health Sciences, Makerere University, Kampala, Uganda; 4 Department of Medical Biochemistry and Microbiology, Zoonosis Science Center, University of Uppsala, Uppsala, Sweden; 5 Clinical Microbiology and Hospital Hygiene, Uppsala University Hospital, Uppsala, Sweden; 6 Sydney Institute for Infectious Diseases, School of Life and Environmental Sciences and School of Medical Sciences, the University of Sydney, Sydney, Australia; 7 Medical and Molecular Laboratory, College of Health Sciences, Makerere University, Kampala, Uganda; 8 Uganda Virus Research Institute, Entebbe, Uganda; 9 Department of Obstetrics and Gynaecology, Kawempe National Referral Hospital, Kampala, Uganda; 10 Department of Obstetrics and Gynaecology, School of Medicine, College of Health Sciences, Makerere University, Kampala, Uganda; 11 Department of Pathology, University of Cambridge, Tennis Court Road, Cambridge, UK

**Keywords:** Pre-eclampsia, Genetic variations, Vascular endothelial growth factor, Single nucleotide polymorphism, Genetic biomarkers, Sanger sequencing, 3’ Untranslated region, Africa, Uganda

## Abstract

**Background:**

Vascular endothelial growth factor A (VEGFA) is a major angiogenic factor that plays an important role in the formation of blood vessels during embryonic development. VEGFA has been implicated in the pathophysiology of pre-eclampsia (PE), since pre-eclamptic women present with reduced levels of free circulating VEGFA. The 3’ untranslated region (3’-UTR) of the VEGFA gene consists of elements that regulate the transcription and hence expression of the VEGFA protein in circulation. Hence it is suggested that variations thereof could underlie the reduced VEGFA levels observed in pre-eclamptic women. The purpose of this study was to investigate presence of the + 936C/T polymorphism, a common single nucleotide polymorphism (SNP) in the 3’-UTR of the VEGFA gene, and determine its association with PE among pregnant women in Uganda.

**Results:**

There was no significant difference observed in the allele and genotype frequencies of the + 936C/T 3’ UTR-VEGFA polymorphism between pre-eclamptic and normotensive pregnant women (*P* > 0.05). Additionally, there was no significant difference in the median plasma levels of free VEGFA among women with the wild type, CT and TT genotypes of the + 936C/T VEGFA polymorphism (median = 0.84 pg/mL (IQR = 0.39–1.41) Vs 1.05 (0.61–1.18) Vs 1.05 (1.05–1.05) respectively, *p*-value = 0.7161).

**Conclusions:**

These study findings indicate that the + 936C/T 3’ UTR-VEGFA polymorphism had no significant association with increased susceptibility to PE among women in Uganda. Further studies with a larger sample size are recommended.

**Supplementary Information:**

The online version contains supplementary material available at 10.1186/s12864-023-09213-8.

## Background

Pre-eclampsia (PE) is a pregnancy complication that is diagnosed after 20 weeks of gestation in a previously normotensive woman, and is characterised by new onset of hypertension, and proteinuria [1]. PE is a major cause of fetal, new-born and maternal morbidity and mortality, affecting 5–7% of pregnant women worldwide [2]. The burden of PE is higher among women of African ancestry as evident by the high attributable maternal mortality rate in sub-Saharan Africa and among women of African ancestry elsewhere in the world [3, 4].

The pathogenesis of PE remains unclear, although vascular endothelial cell injury and dysfunction, and placental ischemia remain core features of the syndrome [5]. Vascular endothelial growth factor A (VEGFA) is a major angiogenic factor that facilitates endothelial cell differentiation, proliferation, migration and invasion, as well as regulates formation and development of blood vessels. VEGFA also plays a key role in trophoblast cell development and function, and is required during placental vascularization to enable sufficient supply of blood to the growing fetus [6]. In fact, reduced expression of VEGFA during placental and embryonic development can lead to embryonic deformities and death [7]. PE is accompanied with an aberrant balance of circulating angiogenic factors, including reduced bioavailability of free VEGFA which is linked to the vascular damage [8, 9]. Low VEGFA levels in pre-eclamptic women are partly attributed to excess release of soluble fms-like tyrosine kinase 1 (sFlt-1) from the placenta, which acts as a decoy receptor, binding free VEGFA in the maternal circulation [10].

Human VEGFA is encoded by the VEGFA gene located on the chromosome 6 p21.3, and consists of 8 exons and 7 introns within a coding region of 14 kb. The VEGFA gene is highly polymorphic with several single nucleotide polymorphisms (SNPs) detected in the untranslated and promoter regions [11, 12]. The 3’ untranslated region (3’-UTR) of the VEGFA gene consists of regulatory elements where binding of transcriptional factors (such as hypoxia induced proteins) effects changes in expression of VEGFA. It is hypothesized that variations within the 3’UTR of the VEGFA gene may result into individual differences in the expression and hence circulating levels of VEGFA [13]. A study by Renner et al. [13] identified a + 936C/T 3’ UTR-VEGFA polymorphism (rs3025039), a common SNP in the 3’UTR of the VEGFA gene, as associated with reduced circulating VEGFA levels. Indeed, in some studies, polymorphisms in the VEGFA gene were associated with dysregulated expression of VEGFA, leading to increased susceptibility to PE [13–15]. The + 936C/T polymorphism was later identified to increase susceptibility to PE among Korean women [16].

Currently in Uganda and the rest of sub-Saharan Africa, less is known about the + 936C/T 3’ UTR-VEGFA polymorphism and its association with PE. Uganda is faced with a high burden of PE, leading to about 6% of the 336 estimated maternal deaths per 100,000 live births per year [17]. Knowledge of genetic biomarkers could improve quality of antenatal care through better risk stratification and early screening of pregnancy complications including PE, hence reducing its burden [18, 19]. In this study therefore, we investigated the association between the + 936C/T 3’ UTR-VEGFA polymorphism and PE among women in Uganda.

## Results

### Baseline characteristics of study participants

The socio-demographic and clinical characteristics of the study participants are summarized in Tables 1 and 2 respectively. There was no significant difference in the maternal and gestational between cases and controls. All study participants were Black Africans by self-report, most of whom were married, and majority in the control group having attained up to secondary level education. The number of participants with a family history of PE, family history of hypertension, and diagnosis with hypertension in the previous pregnancy, was significantly higher among cases as compared to the control group (*p*-values; ˂0.0255, < 0.0034 and 0.0005 respectively).

**Table 1:** Socio-demographic characteristics of study participants

Variable	Cases (*N* = 125)	Controls (*N* = 125)	*P*-values
**Maternal age (years)**			
18–22	43 (34.4)	43 (34.4)	0.9996
23–27	39 (31.2)	39 (31.2)	
28–32	30 (24.0)	30 (24.0)	
33–37	11 (8.8)	11 (8.8)	
38–42	2 (1.6)	2 (1.6)	
**Marital status**			
Married	107 (85.6)	111 (88.8)	0.5701
Single	18 (14.4)	14 (11.2)	
**Level of Education**			
None	0 (0.0)	1 (0.8)	0.2246
Lower primary (1–4)	5 (4.0)	4 (3.2)	
Upper primary (5–7)	37 (29.6)	31 (24.8)	
Lower secondary (1–4)	43 (34.4)	61 (48.8)	
Upper secondary (5–6)	13 (10.4)	10 (8.0)	
Tertiary/university	27 (21.6)	18 (14.4)	
**Smoking**			
Has never smoked	123 (98.4)	125 (100)	0.3649
Stopped during index pregnancy	1 (0.8)	0 (0.0)	
Smoked during index pregnancy	1 (0.8)	0 (0.0)	
**Alcohol consumption during current pregnancy**			
No	114 (91.2)	117 (93.6)	0.7930
Yes	10 (8.0)	8 (6.4)	
Missing data	1 (0.8)	0 (0.0)	

*n* number

**Table 2:** Clinical characteristic of study participants

Variable	Cases (*N* = 125)	Controls (*N* = 125)	*P*-values
**Gestational age (weeks)**			
20–28	11 (8.8)	11 (8.8)	0.9986
29–33	30 (24.0)	30 (24.0)	
34–37	36 (28.8)	36 (28.8)	
38–42	48 (38.4)	48 (38.4)	
**Family history of Diabetes Mellitus**			
No	105 (84.0)	112 (89.6)	0.2623
Yes	20 (16.0)	13 (10.4)	
**Family history of Pre-eclampsia**			
No	116 (92.8)	122 (97.6)	**0.0255**
Yes	9 (7.2)	1 (0.8)	
Missing data	0 (0.0)	2 (1.6)	
**Family history of Hypertension**			
No	78 (62.4)	100 (80.0)	**0.0034**
Yes	47 (37.6)	25 (20.0)	
**HIV status**			
Positive	3 (2.4)	9 (7.2)	0.1391
Negative	122 (97.6)	116 (92.8)	
**First Pregnancy**			
No	71 (56.8)	79 (63.2)	0.3662
Yes	54 (43.2)	46 (36.8)	
**Diagnosis with Hypertension in previous pregnancy**			
No	56 (44.8)	77 (61.6)	**0.0005**
Yes	14 (11.2)	1 (0.8)	
Not applicable	55 (44.0)	47 (37.6)	
**Type of Pregnancy**			
Singleton	115 (92.0)	120 (96.0)	0.1250
Multiple	6 (4.8)	1 (0.8)	
Missing data	4 (3.2)	4 (3.2)	

*n* number

### *Association of the* + *936C/T 3’ UTR-VEGFA gene polymorphism with susceptibility to PE*

**Fig. 1:**
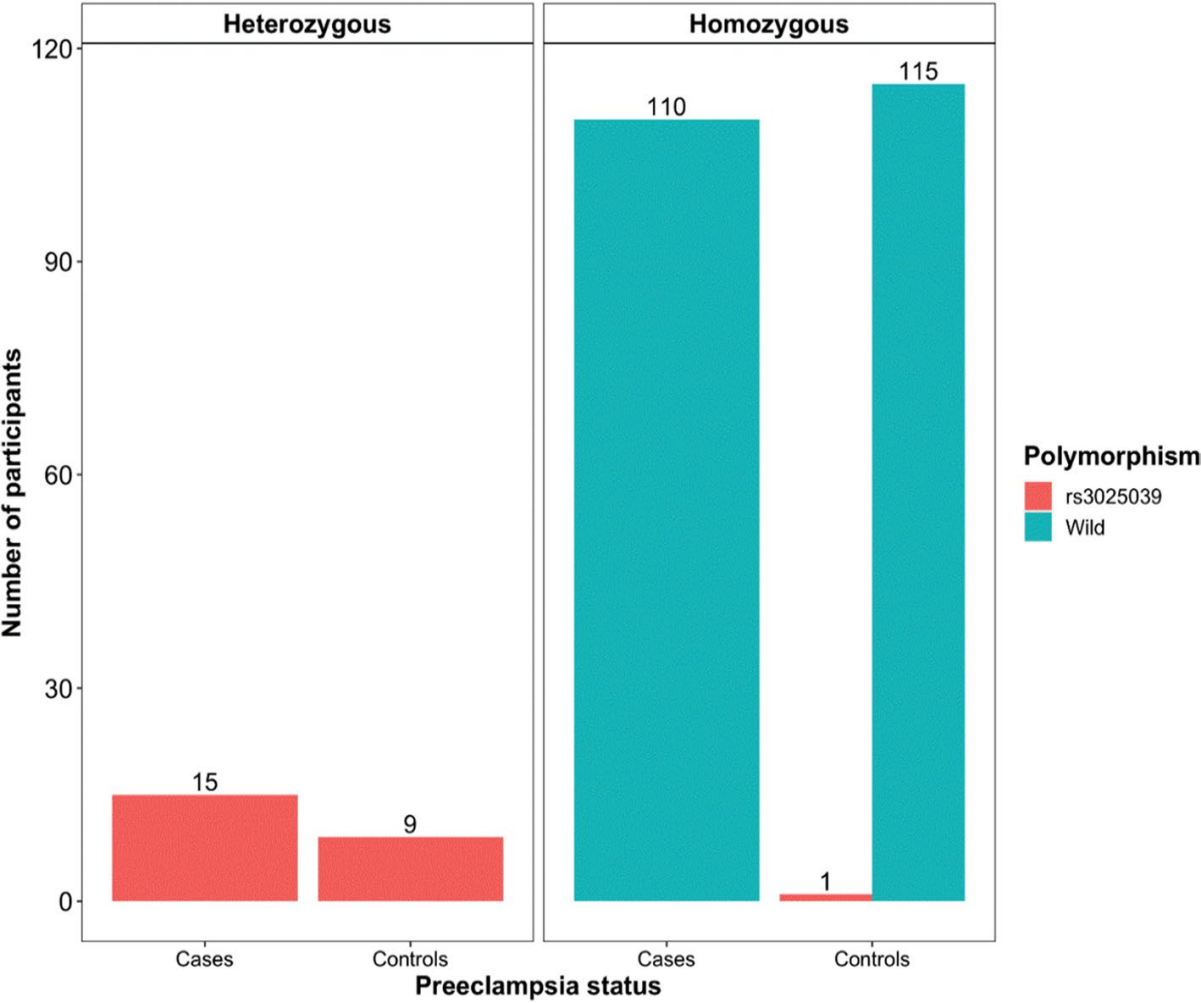
Distribution of the rs3025039 SNP variations among cases and controls. The number of participants with a + 936C/T (rs3025039) variation was higher among cases compared to controls. The wild group indicates participants with the wild type genotype of the rs3025039 SNP

Among all study participants, 225 had the homozygous wild type variant of the + 936C/T SNP, whereas 24 were identified to be heterozygous (CT genotype), and one homozygous (TT genotype) (Fig. 1). To determine whether the + 936C/T 3’ UTR-VEGFA polymorphism is associated with susceptibility to PE, univariate and multivariate conditional logistic regression analysis was performed (Table 3). Clinical and demographic variables with a biological plausibility to PE, and those with *p*-value ≤ 0.2 in the univariate analysis were considered for multivariate analysis. These included; Alcohol consumption, family history of Diabetes mellitus, family history of PE, family history of hypertension, HIV status, diagnosis with hypertension in previous pregnancy, type of pregnancy and Marital status. Findings indicate that presence of the 936 T allele in the 3’UTR of the VEGFA gene is associated with about 2 times increased likelihood of developing PE, however this was not statistically significant (aOR, 1.78 (95% CI, 0.65–4.91), *p*-value = 0.2640). The number of participants with family history of hypertension was higher among cases as compared to controls across all genotypes of the + 936C/T polymorphism (Supplementary Figure S1). However, after Bonferroni correction, family history of hypertension was not significantly associated with PE (aP-value = 0.1109).

**Table 3:** Bivariate and multivariate analysis for association of the + 936C/T 3’ UTR VEGF gene polymorphism with PE

Variable	Cases *N* = 125 n(%)	Controls *N* = 125 n(%)	Crude OR (95% CI)	*P*-value	Adjusted OR (95% CI)	*P*-value	Adjusted *P*-value
** + 936C/T polymorphism**							
Present	15 (12.0)	10 (8.0)	1.56 (0.67–3.59)	**0.3010**	1.78 (0.65–4.91)	0.2640	1.0000
Absent	110 (88.0)	115 (92.0)	1		1		
**HIV status**							
Positive	3 (2.4)	9 (7.2)	0.33 (0.09–1.231)	**0.0994**	0.38 (0.09–1.52)	0.1699	1.0000
Negative	122 (97.6)	116 (92.8)	1		1		
**Alcohol consumption**							
Yes	10 (8.0)	8 (6.4)	1.29 (0.48–3.45)	0.6180	–		
No	114 (91.2)	117 (93.6)	1				
Missing data	1 (0.8)	0 (0.0)					
**Family history of PE**							
Yes	9 (7.2)	1 (0.8)	9.00 (1.14–71.04)	**0.0371**	8.49 (1.00–71.76)	**0.0520**	0.3120
No	116 (92.8)	122 (97.6)	1		1		
Missing data	0 (0.0)	2 (1.6)					
**Family history of hypertension**							
Yes	47 (37.6)	25 (20.0)	2.57 (1.39–4.77)	**0.0027**	2.37 (1.12–5.01)	**0.0185**	0.1109
No	78 (62.4)	100 (80.0)	1		1		
**Diagnosis with hypertension in previous pregnancy**							
Yes	14 (11.2)	1 (0.8)	11.00 (1.42–85.2)	**0.0217**	–		
No	56 (44.8)	77 (61.6)	1				
Missing data	55 (44.0)	47 (37.6)					
**First pregnancy**							
Yes	54	46	1.53 (0.80–2.94)	0.2000	–		
No	71	79	1				
**Type of pregnancy**							
Multiple	6 (4.8)	1 (0.8)	6.00 (0.72–49.84)	**0.0971**	7.25 (0.84–62.94)	0.0722	0.4334
Singleton	115 (92.0)	120 (96.0)	1		1		
Missing data	4 (3.2)	4 (3.2)					
**Family history of diabetes mellitus**							
Yes	20 (16.0)	12 (9.6)	1.64 (0.77–3.47)	**0.1980**	0.99 (0.36–2.76)	0.9910	
No	105 (84.0)	113 (90.4)	1		1		
**Marital status**							
Married	107 (85.6)	111 (88.8)	1.33 (0.63–2.82)	0.4510	–		
Single	18 (14.4)	14 (11.2)	1				

*n* number. Missing data was regarded as missing completely at random due to unavailability of required values on patient files. Adjusted *P*-value, Bonferroni correction

### *Genotypes and allele frequencies of the* + *936C/T 3’ UTR-VEGFA gene polymorphism between women with pre-eclampsia and the controls, and susceptibility to PE*

We further investigated the genotype and allele frequencies of the + 936C/T 3’ UTR-VEGFA polymorphism, and their association to PE susceptibility (Table 4). All loci were in Hardy–Weinberg equilibrium in both the cases and the control group (*P* > 0.05). We observed a higher frequency of the T allele and the CT genotype of the + 936C/T polymorphism among cases compared to controls, however this was not statistically significant (*p*-values; 0.5465 and 0.2829 respectively). Only one individual among all study participants was identified as homozygous genotype TT for the 936C/T polymorphism.

**Table 4:** Comparison of genotypic and allelic frequencies of the + 936C/T 3’ UTR-VEGFA polymorphism among cases and controls

	**Cases *N* = 125**	**Controls *N* = 125**	**HWE**	**OR (95%CI)**	***P*-value**
** + 936C/T polymorphism**					
**Genotypes**					
CC n(%)	110 (88.0)	115 (92.0)		1	ref
CT n(%)	15 (12.0)	9 (7.2)		0.57 (0.21–1.47)	0.2829
TT n(%)	0 (0.0)	1 (0.8)	1	NA	NA
**Alleles**					
C n(%)	235 (94.0)	239 (95.6)		1	ref
T n(%)	15 (6.0)	11 (4.4)		0.72 (0.29–1.72)	0.5465

*HWE* Hardy–Weinberg equilibrium, *n* number

### *Plasma VEGFA levels and the* + *936C/T 3’ UTR-VEGFA SNP genotypes*

**Fig. 2:**
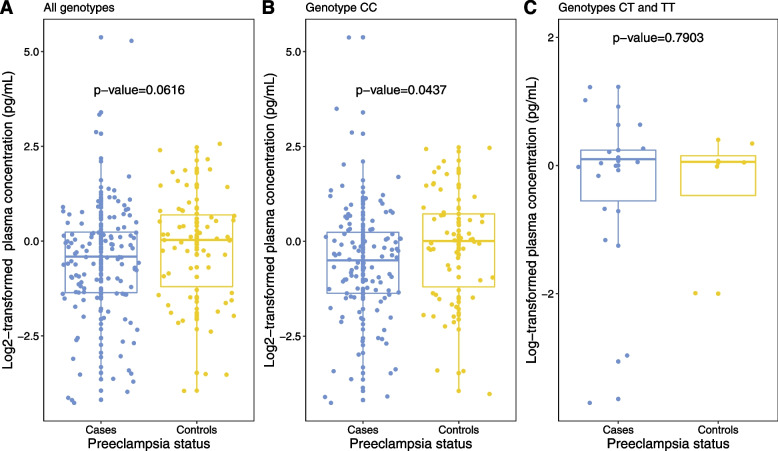
Distribution of free circulating VEGFA plasma levels among genotypes of the + 936C/T polymorphism. **A** Logarithm of the median plasma levels of VEGFA in cases versus controls (All genotypes). **B** Logarithm of the median plasma levels of VEGFA among cases and controls with only the wild type CC genotype. **C** Logarithm of the median plasma levels of VEGFA among cases and controls with the CT and TT genotypes

Secondarily, we investigated the difference in the plasma levels of free circulating VEGFA by each of the + 936C/T SNP genotypes among the cases and controls (Table 5). The concentration of VEGFA was log transformed to the base of two prior to this analysis. A total of 180 samples were assayed for the concentration of free plasma VEGFA (including 122 cases and 58 controls), among which only 18 were found to have the + 936C/T SNP, 17 of which were heterozygous (CT genotype) and one homozygous (TT genotype). The concentration of plasma free VEGFA was significantly lower among the cases as compared to controls (median = 0.76 pg/mL (IQR = 0.39–1.18) Vs 1.02 pg/mL (0.44–1.62) respectively, *p*-value = 0.0616) (Fig. 2). Cases with the CC wild genotype had significantly lower VEGFA plasma levels as compared to controls with the same genotype (median = 0.8 pg/mL (IQR = 0.38–1.18) Vs 1.0 pg/mL (0.44–1.65) respectively, *p*-value = 0.0437). Among only samples with the CT genotype, the plasma levels of free VEGFA were higher among cases compared to controls. Median concentration of free plasma VEGFA was higher among individuals with the heterozygous and homozygous genotypes, as compared to those with the wild type +936C/T polymorphism. These findings however lacked statistical significance.

**Table 5:** Comparison of plasma VEGFA levels between genotypes of the + 936C/T 3’ UTR-VEGFA polymorphism

Status	Genotype	Median in pg/mL (IQR)	Total samples	*P*-value
Cases	Wild CC	0.71 (0.38–1.18)	108	0.0437
Controls	Wild CC	1.00 (0.44–1.65)	54	
Cases	+ 936C/T _het	1.07 (0.70–1.18)	14	0.8499
Controls	+ 936C/T _het	1.03 (0.64–1.18)	3	
Combined cases and controls	Wild	0.84 (0.39–1.41)	162	0.7161
Combined cases and controls	+ 936C/T _het	1.05 (0.61–1.18)	17	
Combined cases and controls	+ 936C/T _hom	1.05 (1.05–1.05)	01	

Wild; study participants without of the + 936C/T polymorphism (CC genotype), *het* heterozygous (CT genotype), *hom* homozygous (TT genotype)

## Discussion

Pre-eclampsia (PE) is a complex pregnancy complication with multiple genetic links [20]. VEGFA, a major angiogenic factor that regulates vascular formation and function, as well as placental development, plays a crucial role in the pathophysiology of PE [21]. Previously, various studies among pregnant women have indicated a decrease in plasma VEGFA levels as one of the features for PE [8, 22, 23]. Genetic mechanisms are suggested to underlie reduced expression of circulating VEGFA, implicating variations/mutations in the VEGFA gene [24]. In this study we describe the detection of the +936C/T polymorphism; a common SNP variation in the 3’ UTR of the VEGFA gene, and its association with susceptibility to PE. We further describe the plasma levels of free circulating VEGFA by each of the genotypes of the +936C/T polymorphism among both pre-eclamptic and normal pregnant women.

Our findings indicate that the + 936C/T 3’ UTR-VEGFA polymorphism was associated with about 2 times increased likelihood of developing, however this was not statistically significant. Hence, no significant association was found between the + 936C/T polymorphism and increased susceptibility to PE among the women attending Mulago National Referral Hospital in Uganda. We further observed no significant difference in the genotype and allele frequencies of the + 936C/T 3’ polymorphism between the cases and controls. Our findings are similar to a study done among Sinhalese women that indicated no significant difference in the genotype frequencies of the + 936C/T polymorphism among cases with PE and normotensive pregnant women. That is, there was no significant association between the + 936C/T 3’ UTR-VEGFA polymorphism and risk of developing PE [25]. However, contrary to our findings, a study by Shim et al. [16] found that carriage of the + 936 T allele was independently associated with increased susceptibility to PE among Korean women. The frequency of the + 936C/T 3’ UTR-VEGFA polymorphism among the Korean women was significantly higher among cases as compared to controls. In another study, the CT, CT + TT genotypes, and T allele of the + 936C/T were risk factors for pre-eclampsia in Sudanese women [26]. This inconsistency could be attributed to ethnic differences between the study populations, and environmental, immunological and lifestyle factors that interactively contribute to the multifactorial complex of the PE syndrome [27]. Other previous studies have similarly indicated a link between the + 936C/T 3’ UTR-VEGFA polymorphism and increased risk of developing PE in other populations such as Asians, Caucasians and Latinos [16, 28–30].

We further investigated the difference in the concentration of plasma free circulating VEGFA by each of the + 936C/T genotypes among the cases and controls. Overall, the pre-eclamptic women had significantly lower plasma free VEGFA levels compared to the control group. This is similar to findings from previous studies [8, 22, 31]. In this study, we found that participants with the CT and TT genotypes of the + 936C/T polymorphism had higher VGEF levels compared to participants with the wild type CC genotype, however, this difference was not statistically significant. Contrary to these findings, a study by Procopciuc LM et al., [32] indicated that carriers of the 936 T allele among mothers affected by PE had significantly reduced levels of circulating VEGFA. Presence of the 936 T allele is associated with reduced ability to upregulate VEGFA production hence enhancing development of PE [33].

Among other risk factors linked with susceptibility to PE in this study, multivariate analysis indicated that women with a family history of hypertension were more likely to develop PE in the current pregnancy. However, after correcting for multiple testing using Bonferroni correction, these findings were not statistically significant. Our findings then differ from previous literature that shows a strong link between family history of hypertension and PE. In a study by Bezerra et al. [34], the risk of developing PE was two times higher in a pregnant woman whose sister had an account of hypertension (OR 2.60, 95% CI 1.60–4.21, *p* < 0.001). The risk of developing PE was even higher (3 times) among pregnant women who had both a mother and sister with hypertension (OR 3.65, 95% CI 1.65–8.09, *p* = 0.001).

Still at multivariate analysis, we further observed a borderline association between family history of PE and susceptibility to PE, which was non-significant after correction for multiple testing. However, previously, it is well recognised that family history of PE is associated with increased likelihood of developing the syndrome in a current pregnancy [35, 36]. In a study done among women in Taiwan, mothers with a sororal history of PE similarly had a higher risk for pre-eclampsia compared to those without (relative risk; 2.6, CI; 2.41–2.80) [36].

## Conclusions and recommendations

This is one of the few studies investigating genetic factors associated with PE among women in the Ugandan population. A previous study in this population revealed that a *KIR 2DS5* gene locus was associated with protection against PE [37]. Identification of key genetic markers of PE is crucial especially among the highly burdened African populations which double with high genetic diversity.

In conclusion, there was no significant association between the + 936C/T 3’ UTR-VEGFA polymorphism and increased likelihood of developing PE, and no significant difference in the circulating free VEGFA levels by each of the genotypes of the + 936C/T polymorphism. Despite the fact that our sample size estimation was deliberate to afford 80% power of the study, we recommend a bigger sample sized study to ensure accuracy of these results. Nonetheless, we believe that our results increase the body of knowledge on factors associated with pre-eclampsia among women in Uganda.

Furthermore, the VEGFA gene has multiple variations, which warrants further studies targeting other SNP variations and their haplotype effect towards susceptibility to PE. And given the fact that PE is a heterogeneous syndrome with several genes implicated in its pathophysiology, investigations on how these multiple genes interact are much required towards defining the complex mechanism of PE and identification of genetic markers of susceptibility.

## Methods

### Study population and procedures

In this study, a matched case–control design was utilized to enroll pregnant women aged 18–42 years, at 20 or more weeks of gestation, from Mulago National referral hospital, Kampala, Uganda. Cases were defined as pregnant women presenting with new onset hypertension; including increased systolic blood pressure (BP) ≥ 140 mmHg and diastolic BP ≥ 90 mmHg measured on 2 occasions at least 4 h apart, and proteinuria ≥ + 1 on urine dipstick test [17]. Controls were defined as healthy pregnant women presenting with normal systolic BP ˂ 140 mmHg and diastolic BP˂ 90 mmHg on two occasions at least 4 h apart, with trace or negative urine protein. Controls were matched to identified cases by maternal and gestational ages at a ratio of 1:1 using the following categories; 18–22, 23–27, 28–32, 32–42 years, and 20–28, 29–33, 34–37, 38–43 weeks of gestation. These included a total of 125 women with PE as cases and 125 pregnant women without PE as controls, enrolled from the maternity ward and the outpatient antenatal clinics respectively, from March to October 2019. Women with confirmed fetal abnormalities and pre-existing pathologies (including; diabetes mellitus, cardiovascular disease, chronic renal disease), and those who had received blood transfusion in the previous 3 months were excluded from study participation.

Participant Interviews were conducted using a study designed data tool to obtain socio-demographic and clinical characteristics of study participants; including maternal and gestational age, marital status, level of education, social history (smoking and alcohol consumption), family medical history (diabetes mellitus, PE and hypertension) and personal medical history (HIV status, parity, type of pregnancy and diagnosis with hypertension in a previous pregnancy). Six milliliters (6 mL) of ethylenediaminetetraacetic acid (EDTA) anti-coagulated blood were collected from each study participant via the antecubital vein by a trained study midwife/nurse. Four milliliters (4 mL) of the blood sample were used to extract deoxyribonucleic acid (DNA). Platelet poor plasma was obtained from the rest 2 mL of blood sample using methods previously described [8].

### Extraction of DNA

DNA extraction was done using the Qiagen whole blood DNA extraction kit (QIAamp DNA Blood Midi Kit), following the manufacturer’s instructions. Briefly, for each participant sample, designated kit buffers were mixed with the sample to lyse the blood cells, stabilize nucleic acids, and enhance selective DNA adsorption to the QIAamp membrane. Alcohol was added to the lysates which were thereafter loaded onto the QIAamp spin column. Wash buffers were used to remove impurities from the spin column QIAamp membrane, and pure ready-to-use DNA eluted in low-salt AE buffer. Extracted DNA was quantified using a nanodrop spectrophotometer (Thermo Scientific™ NanoDrop 2000c) and stored temporarily at -20 °C.

### Polymerase chain reaction amplification, Gel electrophoresis and SNP Genotyping

DNA amplification was achieved using conventional polymerase chain reaction (PCR) assay, targeting a specific region within the 3’UTR of the VEGFA gene. Specific PCR primers were designed using the NCBI web-based primer BLAST tool as described by Jian Ye et al., [38]. The designed PCR primer sequences were synthesized by the Eurofins Genomics company (Eurofins Genomics AT GmbH, Viehmarktgasse 1B/Büro 2, 1030, Vienna, Austria), and these comprised the following sequences;

Forward, 5ˈ- TTTGTTTTCCATTTCCCTCAGAT -3ˈ.

Reverse, 5ˈ- CCAACTCAAGTCCACAGCAGTC -3ˈ—targeting a 545 bp product within the 3’ UTR of the VEGFA gene. Each PCR assay was performed in a 50 μL reaction volume containing 5 μL of genomic DNA (50-100 ng/μL), 1 μL of 10 mM Deoxynucleotide (dNTP) solution Mix (NEW ENGLAND BioLabs), 1.5 μL of each primer (10 pmol/μL), 0.125 μL of DreamTaq Hot Start DNA polymerase (5 U/μL, Thermo Scientific, Lithuania), 5 μL of 10X DreamTaq buffer (Thermo Scientific, Lithuania), and 35.875 μL of Nuclease free water. The PCR reactions were run on a conventional thermal cycler (BIO-RAD, T100 Thermal Cycler) using the following program; Initial denaturation at 95 °C for 3 min followed by 35 cycles of denaturation at 95 °C for 30 s, annealing at 62.2 °C for 1 min and extension at 72 °C for 1 min, then a final extension at 72 °C for 10 min and the holding step at 4 °C.

**Fig. 3:**
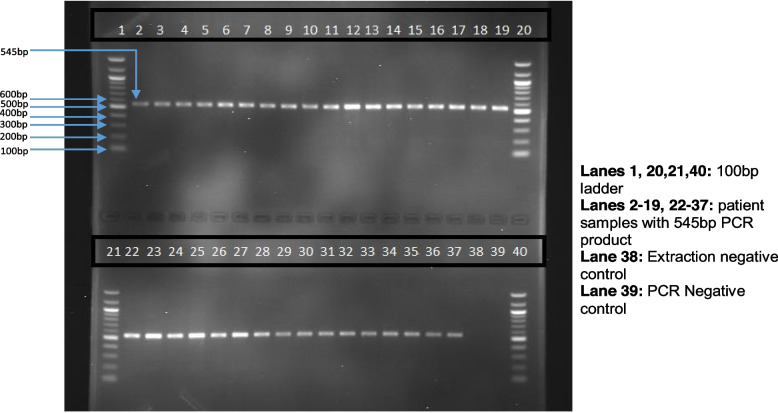
PCR amplification of 3’UTR- VEGFA. Agarose gel electrophoretogram of a 545 bp sequence within the 3’UTR of the VEGFA gene amplified by PCR from different human samples

Detection of PCR products was achieved using gel electrophoresis on a 1.5% agarose gel (Fig. 3). Gel viewing was done using ultraviolet light in a UV transilluminator (UVP Biodoc-It Uv Transilluminator Imaging System).

The PCR amplicons were purified, and sequenced at ACTG Inc. (Wheeling II, USA). Sanger sequencing was employed to sequence the purified amplicons, using an ABI Prism 3100 Sequencer (Applied Biosystems, Foster City, CA, USA). All quality control samples had a 100% rate of agreement.

### Plasma VEGFA assay

Assessment of plasma free VEGFA levels was done using Magnetic luminex performance assay (Human Angiogenesis Premixed Kit A; R&D Systems, a bio-techne brand), following by the manufacturer’s instructions. Analysis of Luminex data was done using Milliplex analyst software. Due to their unreliability, VEGF concentrations below 0.2 pg/mL (the border of reliability as suggested by the = Milliplex analyst software) were divided by the factor 2 in order to prevent spurious positive associations.

Inter-assay coefficient of variation was 10.03%. Part of the VEGFA results presented in this article have been published (8).

### Data analysis

#### Sample size estimation

In reference to a study by Shim et al. [16], we assumed a probability of exposure of 15.1% to the + 936C/T 3’ UTR-VEGFA polymorphism among normotensive pregnant women in the control group, an odds ratio of exposure among the pre-eclamptic relative to the normotensive of 2.45 and 2% loss of samples, for a one control per case, at least 125 cases hence 125 controls are required to test the hypothesis that the odds ratio is equal to 1 considering power of 80% and 5% type 1 error probability associated with the test of the hypothesis. Sample size calculation was done using the epiR package [39].

#### Bioinformatics analysis

**Fig. 4:**
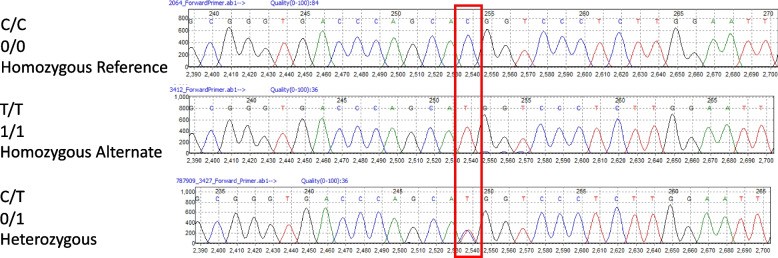
Chromatogram. Showing + 936C/T SNP mapped in the 3’UTR of the human VEGFA gene

Using a sanger sequencing data analysis tool; tracy v0.5.9 [40], the reference human genome (GRCh37) was indexed. The obtained sample sequences were then aligned to the reference, variants called and annotated based on the GRCh37 human genome build. The resultant Binary Call Format (BCF) files were converted to Variant Call Format (VCF) files using bcftools v1.8 [41]. The variants were screened for the presence of the + 936C/T (rs3025039) variant using VCFtools v0.1.16 [42]. All this was done using customized bash scripts. Figure 4 indicates sequencing chromatographs showing the rs3025039 SNP mapped in the 3’ UTR of the VEGFA gene.

#### Statistical analysis

Categorical variables were summarized as absolute numbers and proportions. Continuous variables were summarized as medians and corresponding interquartile ranges. Mann–Whitney U-test was used to compare continuous variables and Fisher’s exact test or Chi-square test for comparing categorical variables. The association of the + 936C/T polymorphism with PE was explored using conditional logistic regression. All analyses were performed in R statistical programming environment version 4.0.5. Packages used for analysis include; survival for conditional logistic regression and ggplot2 for visualizing analysis results [43].

## Supplementary Information

**Additional file 1:** **Supplementary Figure S1.** Distribution of family history of hypertension among genotypes of the of the +936C/T polymorphism in cases and controls.

**Additional file 2.**

## Data Availability

The dataset supporting the conclusions of this article is included within the article (see Supplementary information file). The DNA sequence data generated and analysed during the current study is available in GenBank, accession numbers: ON777416—ON777792.

## Abbreviations

aOR: Adjusted odds ratio
BCF: Binary Call Format
BLAST: Basic Local Alignment Search Tool
BP: Blood pressure
DNA: Deoxyribonucleic acid
EDTA: Ethylenediaminetetraacetate
HWE: Hardy–Weinberg equilibrium
NCBI: National Center for Biotechnology Information
OR: Odds ratio
PE: Pre-eclampsia
sFlt-1: Soluble fms-like tyrosine kinase 1
SNP: Single nucleotide polymorphism
UTR: Untranslated region
VCF: Variant Call format
VEGFA: Vascular endothelial growth factor A
mL: Milliliters
µL: Microlitre
pg/mL: Picogram per milliliter
3’: Three prime
