# Supplementary material for: No association of a Vascular endothelial growth factor A (VEGFA) gene polymorphism with pre-eclampsia among pregnant women in Uganda
Source: BMC Genomics. 2023 Mar 20;24:132. doi: 10.1186/s12864-023-09213-8 (PMC10029258; doi:10.1186/s12864-023-09213-8)
Supplement: Supplementary file 1 — Additional file 1: Supplementary Figure S1. Distribution of family history of hypertension among genotypes of the of the +936C/T polymorphism in cases and controls. [file 12864_2023_9213_MOESM1_ESM.pdf]

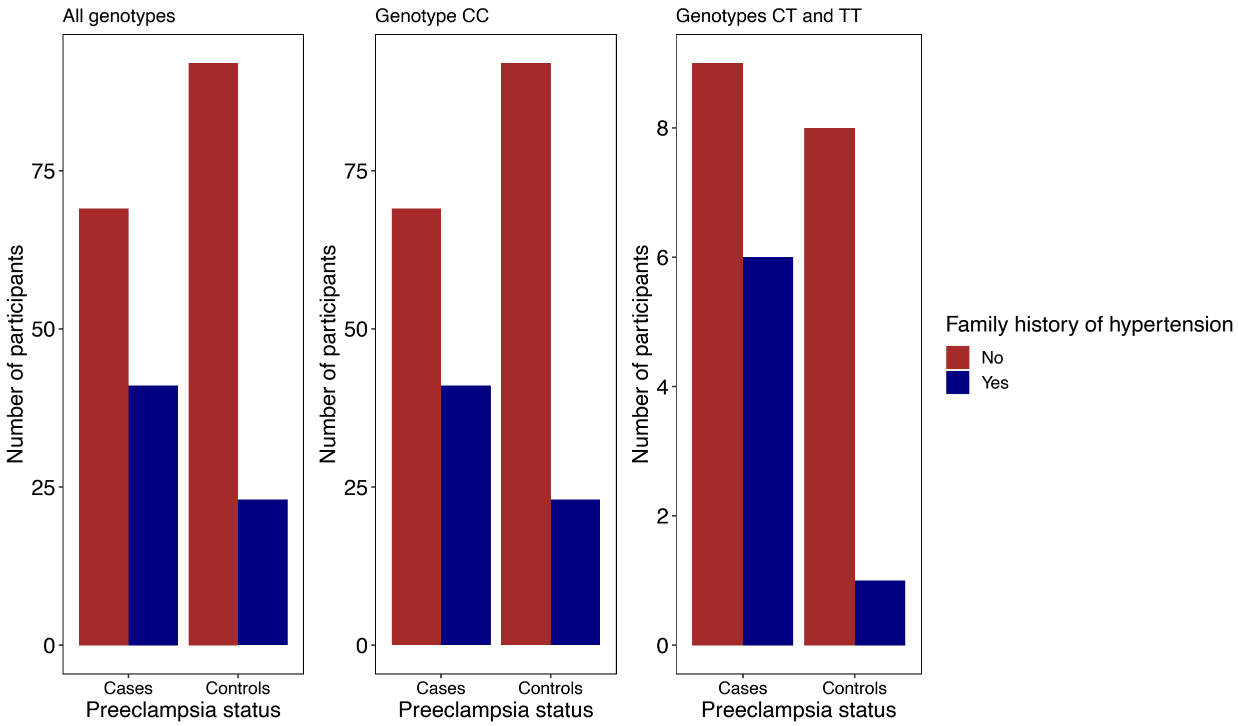

Supplementary Figure S1. Distribution of family history of hypertension among genotypes of the of the +936C/T polymorphism in cases and controls.
